# Administration of Zinc plus Cyclo-(His-Pro) Increases Hippocampal Neurogenesis in Rats during the Early Phase of Streptozotocin-Induced Diabetes

**DOI:** 10.3390/ijms18010073

**Published:** 2017-01-01

**Authors:** Bo Young Choi, In Yeol Kim, Jin Hee Kim, Bo Eun Lee, Song Hee Lee, A Ra Kho, Min Sohn, Sang Won Suh

**Affiliations:** 1 Department of Physiology, College of Medicine, Hallym University, Chuncheon 24252, Korea; bychoi@hallym.ac.kr (B.Y.C.); inyeol@hallym.ac.kr (I.Y.K.); fate0710@hallym.ac.kr (J.H.K.); supsock1126@naver.com (B.E.L.); sshlee@hallym.ac.kr (S.H.L.); rnlduadkfk136@hallym.ac.kr (A.R.K.); 2 Department of Nursing, Inha University, Incheon 22212, Korea; sohnmin@inha.ac.kr

**Keywords:** zinc, zinc plus cyclo-(His-Pro), neurogenesis, hippocampus, diabetes, streptozotocin

## Abstract

The effects of zinc supplementation on hippocampal neurogenesis in diabetes mellitus have not been studied. Herein, we investigated the effects of zinc plus cyclo-(His-Pro) (ZC) on neurogenesis occurring in the subgranular zone of dentate gyrus after streptozotocin (STZ)-induced diabetes. ZC (27 mg/kg) was administered by gavage once daily for one or six weeks from the third day after the STZ injection, and histological evaluation was performed at 10 (early phase) or 45 (late phase) days after STZ injection. We found that the proliferation of progenitor cells in STZ-induced diabetic rats showed an increase in the early phase. Additionally, ZC treatment remarkably increased the number of neural progenitor cells (NPCs) and immature neurons in the early phase of STZ-induced diabetic rats. Furthermore, ZC treatment showed increased survival rate of newly generated cells but no difference in the level of neurogenesis in the late phase of STZ-induced diabetic rats. The present study demonstrates that zinc supplementation by ZC increases both NPCs proliferation and neuroblast production at the early phase of diabetes. Thus, this study suggests that zinc supplemented with a histidine/proline complex may have beneficial effects on neurogenesis in patients experiencing the early phase of Type 1 diabetes.

## 1. Introduction

Type 1 diabetes is reported to make up approximately 5%–10% of the total diabetic population and represents a very significant risk to public health. Cognitive deficits recognized in type 1 diabetes patients include reduced information processing speeds [1,2] and worsening psychomotor function [1,3]. However, the mechanism by which type 1 diabetes patients develop cognitive dysfunction is not clear.

Several studies have demonstrated that new neurons are continuously produced in the rodent brain throughout life [4,5,6], both in the subgranular zone (SGZ) of the hippocampal dentate gyrus (DG) and subventricular zone (SVZ) of the lateral ventricle [7,8]. It is believed that hippocampal neurogenesis plays a role in learning and memory function [9,10]. Our previous studies have demonstrated that zinc, an essential trace element, is involved in hippocampal neurogenesis with or without brain injury. Our lab demonstrated that continuous free zinc release from degenerating DG cells may perpetually produce a signal that drives progenitor cell proliferation and aids the survival of neuroblasts following hypoglycemia [11], epilepsy [12], and traumatic brain injury [13]. In addition, we recently demonstrated that increasing hippocampal vesicular zinc by zinc supplemented with a histidine/proline complex (zinc plus cyclo-(His-Pro) (ZC)) promotes hippocampal neurogenesis under physiological conditions [14].

Therefore, the present study tested the hypothesis that ZC treatment can increase hippocampal neurogenesis in streptozotocin (STZ)-induced diabetic rats. We define the “early phase” as seven days after the STZ-induced diabetic condition and the “late phase” as 42 days after STZ-induced diabetic condition, in rats. We observed three interesting findings. First, progenitor cell proliferation in the hippocampus of diabetic rats showed an increase during the early phase. Second, zinc supplement provided by ZC administration in diabetic rats increased NPC proliferation rate and neuroblast production during the early phase. Third, zinc supplement by ZC administration in diabetic rats showed an increased survival rate of newly generated cells, but no difference in the level of neurogenesis during the late phase. Therefore, the present study suggests that zinc supplementation during the early phase of diabetes may have beneficial effects on hippocampal neurogenesis. However, zinc supplementation during the late phase produced no observable neurogenic effect in the hippocampus.

## 2. Results

### 2.1. ZC Treatment Does Not Affect Body Weight or Blood Glucose Level in Diabetic Rats

Weight change and blood glucose deregulation are the most prominent features of diabetes. Song et al. demonstrated that ZC administration improved body weight control in genetically diabetic rats [15] and decreased blood glucose levels in STZ-induced diabetic rats [16]. Thus, we examined the effects of ZC treatment on body weight and blood glucose level in rats that had undergone STZ-induced diabetes during our experimental period (i.e., early (10 days) or late (45 days) diabetic phase). Rats were given ZC (27 mg/kg, per os (PO)) once per day for one or six weeks. Compared to the sham-operated group, the STZ-injected group showed a decrease in body weight and a rapid increase in blood glucose levels. However, ZC treatment itself induced no weight or blood glucose changes, either in the sham-operated or in the STZ-injected group (Table 1 and Table 2).

### 2.2. Short-Term ZC Treatment Increases the NPCs Proliferation in Diabetic Rats

**Figure 1:**
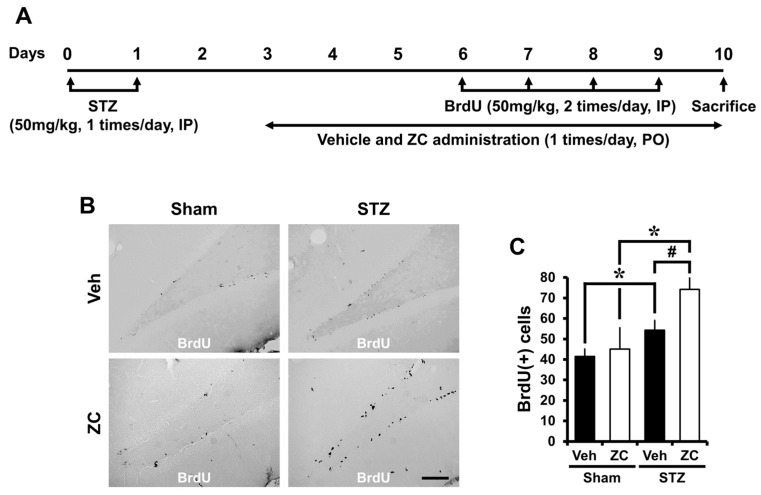

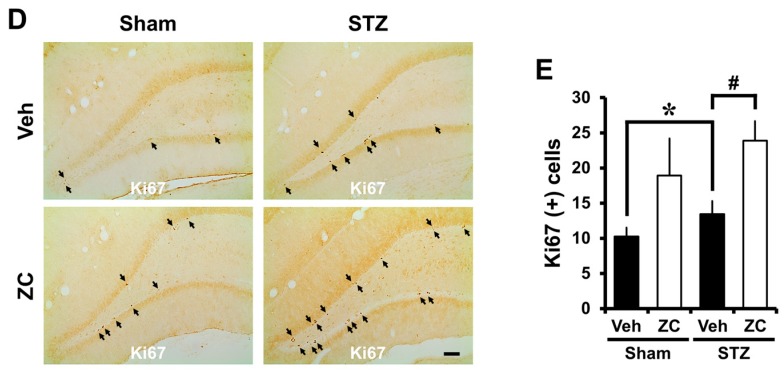
ZC increases proliferation of progenitor cells in the early phase of STZ-induced diabetic rats. (**A**) Experimental procedure in the early phase of STZ-induced diabetic rats. IP: intraperitoneal, PO: per os; (**B**) Bright field photomicrographs show BrdU (+) progenitor cells in the hippocampal dentate gyrus (DG). In the early phase, STZ-injected group showed a significant increase of BrdU (+) cells. ZC treatment by gavage for one week after STZ-induced hyperglycemia further increased BrdU (+) cells. Scale bar = 100 µm; (**C**) Bar graph represents the number of BrdU-positive cells in the subgranular zone (SGZ) of DG. Data are means ± SE, *n* = 7–12 from each group. * *p* < 0.05, versus vehicle-treated sham group; # *p* < 0.05, versus vehicle-treated STZ group; (**D**) Representative photomicrographs show Ki67 (+) cells in the hippocampal DG. The Ki67 (+) cells is indicated by a black arrow. In the early phase, the STZ-injected group showed a significant increase of Ki67 (+) cells. Short-term ZC treatment increased the number of Ki67 positive cells in STZ-induced diabetic rats. Scale bar = 100 µm; (**E**) Bar graph represents the number of Ki67 (+) cells in the SGZ of DG. Data are means ± SE, *n* = 7–12 from each group. * *p* < 0.05, versus vehicle-treated sham group; # *p* < 0.05, versus vehicle-treated STZ group.

To test whether ZC affects progenitor cell proliferation during the early phase of STZ-induced diabetes, rats were sacrificed at one week following daily ZC treatment with or without STZ injection. 5-Bromo-2-Deoxyuridine (BrdU) was intraperitoneally (IP) injected twice per day for four consecutive days from the sixth day following STZ injection. Cellular proliferation was assessed by BrdU and Ki67 immunohistochemistry. We found an increase in the number of cells labeled by both BrdU and Ki67 immunostaining in the SGZ of DG of animals that received STZ injection. In addition, ZC administration after STZ injection significantly increased the number of BrdU and Ki67 positive cells in the SGZ of DG (Figure 1).

**Figure 2:**
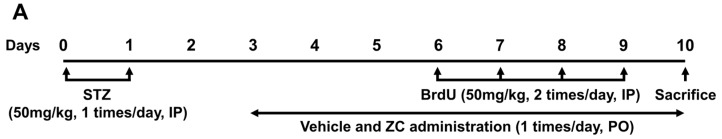

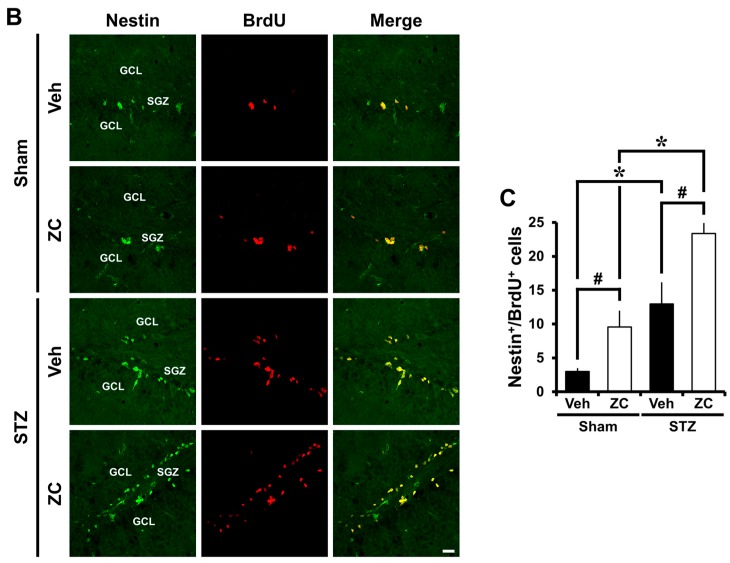
ZC increases neural progenitor cells (NPCs) proliferation in the early phase of STZ-induced diabetic rats. (**A**) Experimental procedure in the early phase of STZ-induced diabetic rats; (**B**) Fluorescent images show Nestin^+^/BrdU^+^ cells. In the early phase, the STZ-injected group showed a significant increase of Nestin^+^/BrdU^+^ cells. Short-term ZC treatment increased the number of Nestin^+^/BrdU^+^ cells in both the sham-operated and the STZ-injected group. Scale bar = 20 µm; (**C**) Bar graph represents the number of Nestin^+^/BrdU^+^ cells in the SGZ of DG. Data are means ± SE, *n* = 5–6 from each group. * *p* < 0.05, versus sham group; # *p* < 0.05, versus vehicle-treated group. GCL: granular cell layer; SGZ: subgranular zone.

Next, we determined whether theses progenitor cells are neural precursors. To this end, double immunofluorescence for BrdU and Nestin, an intermediate filament protein used to identify neural stem cells [17], was conducted. The number of BrdU and Nestin co-labeled cells in the DG was significantly increased after STZ injection. Additionally, the number of BrdU and Nestin co-labeled cells after daily ZC treatment in both the sham-operated and the STZ-injected group was also significantly increased in the DG compared to vehicle-treated rats (Figure 2).

### 2.3. Short-Term ZC Treatment Increases Neuroblast Production in Diabetic Rats

**Figure 3:**
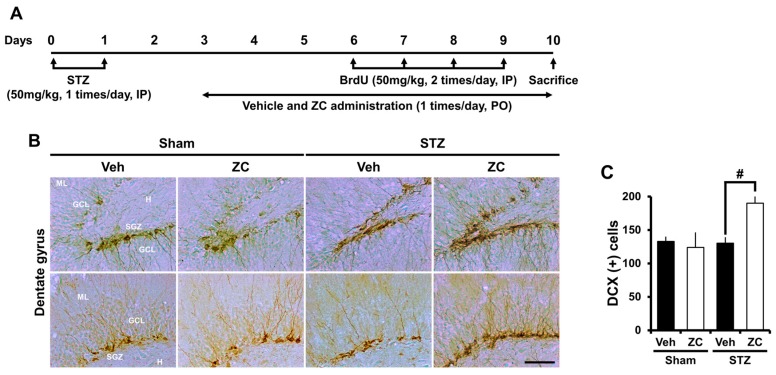
ZC increases DCX-positive cells in the early phase of STZ-induced diabetic rats. (**A**) Experimental procedure in the early phase of STZ-induced diabetic rats; (**B**) Photomicrographs show DCX (+) neuroblasts in the hippocampal DG. The STZ-injected group showed a similar level of DCX expression compared to the sham-operated group. However, ZC treatment by gavage for one week after STZ-induced hyperglycemia significantly increased DCX (+) cells. Scale bar = 100 µm; (**C**) Bar graph represents the number of DCX-positive cells in the SGZ/GCL. Data are means ± SE, *n* = 7–12 from each group. # *p* < 0.05, versus vehicle-treated STZ group. ML: molecular layer; GCL: granular cell layer; SGZ: subgranular zone; H: hilus.

To assess whether ZC influences newly generated immature postmitotic neurons during the early phase of STZ-induced diabetes, rats were sacrificed one week after daily ZC treatment with or without STZ injection. Neuroblast production was evaluated by doublecortin (DCX) immunohistochemistry. In the sham-operated group, the number of DCX positive cells in the DG was similar between vehicle-treated rats and ZC-treated rats. The number of DCX positive cells was not different after STZ injection compared to the sham-operated group. However, ZC-treated rats showed a significantly higher number of DCX positive cells in the DG compared to vehicle-treated rats after STZ injection (Figure 3).

### 2.4. Long-Term ZC Treatment Increases the Survival of BrdU Positive Cells in Diabetic Rat

**Figure 4:**
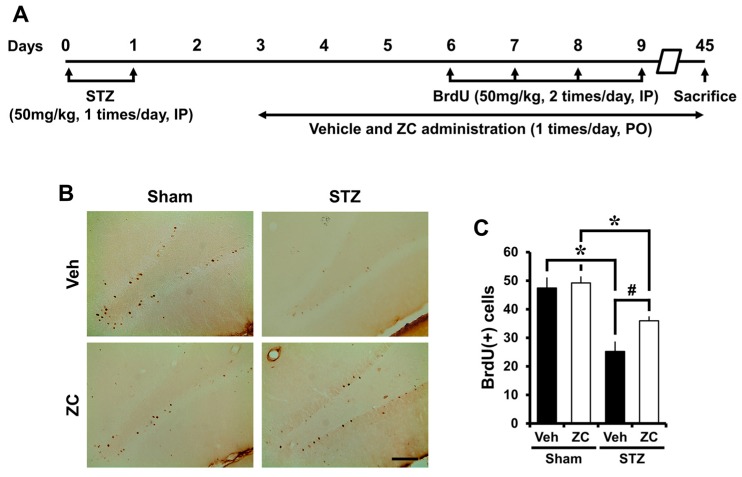
ZC increases survival of BrdU-positive cells in the late phase of STZ-induced diabetic rats. (**A**) Experimental procedure in the late phase of STZ-induced diabetic rats; (**B**) Bright field photomicrographs show surviving newborn cells. In the late phase, the STZ-injected group showed a significant decrease of BrdU (+) cells. However, ZC treatment by gavage for six weeks after STZ-induced hyperglycemia significantly increased BrdU (+) cells. Scale bar = 100 µm; (**C**) Bar graph represents BrdU-positive cells in the SGZ/GCL. Data are means ± SE, *n* = 6–8 from each group. * *p* < 0.05, versus sham group; # *p* < 0.05, versus vehicle-treated STZ group.

To test whether ZC affects survival of newborn cells in the late phase of STZ-induced diabetes, rats were sacrificed six weeks after daily ZC treatment with or without STZ injection. Identical to the early phase, BrdU was intraperitoneally injected twice per day for four consecutive days from the sixth day following STZ injection. Surviving newborn cells, as detected by BrdU immunohistochemistry, were distributed throughout the entire granular cell layer (GCL). Some of those cells had already migrated into the GCL, whereas others were still localized in the SGZ. Although the progenitor cells proliferation rate was remarkably increased 10 days after STZ injection, the number of surviving newborn cells was decreased at 45 days after STZ injection. However, ZC-treated rats showed a significantly higher number of BrdU positive cells in the DG compared to vehicle-treated rats after STZ injection (Figure 4).

### 2.5. Long-Term ZC Treatment Does Not Affect the Neurogenesis in Diabetic Rats

**Figure 5:**
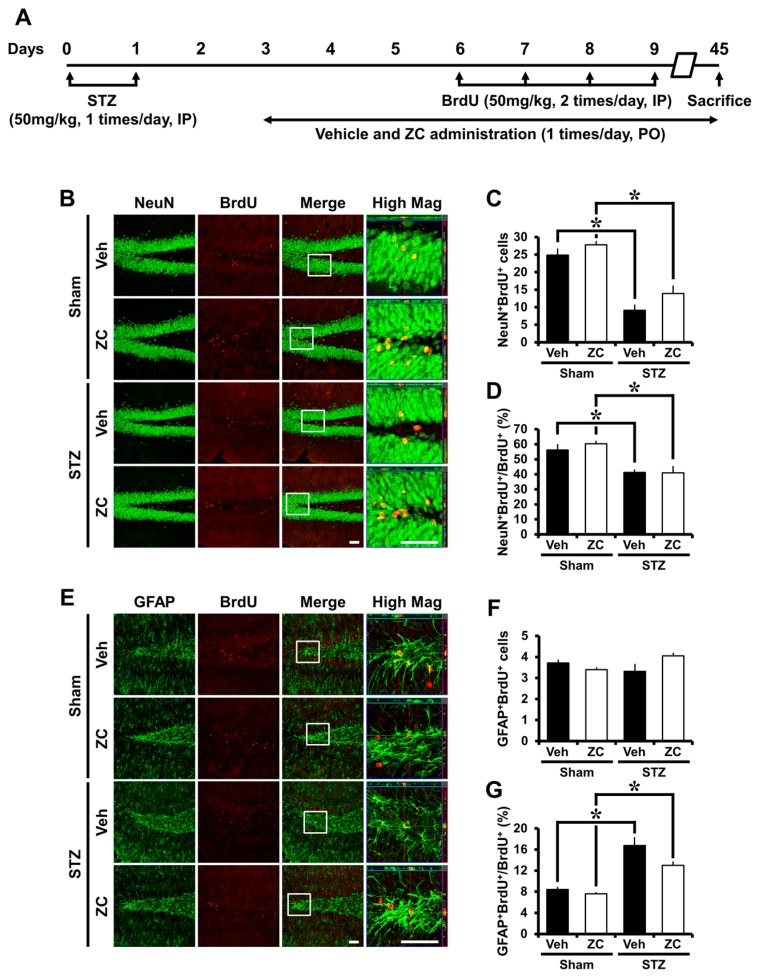
ZC does not affect neurogenesis in the late phase of STZ-induced diabetic rats. (**A**) Experimental procedure in the late phase of STZ-induced diabetic rats; (**B**) Representative images show double-labeling immunofluorescence of BrdU and NeuN positive cells in the hippocampal DG. STZ-injected group showed a significant decrease of BrdU^+^/NeuN^+^ cells. ZC treatment by gavage for six weeks after STZ-induced hyperglycemia showed no difference in the number of BrdU^+^/NeuN^+^ cells. Scale bar = 100 µm; (**C**) Bar graph represents the number of BrdU^+^/NeuN^+^ cells in the SGZ/GCL; (**D**) Bar graph show the proportion of BrdU^+^/NeuN^+^ cells in BrdU^+^ cells of the SGZ/GCL. Data are means ± SE, *n* = 5–8 from each group. * *p* < 0.05, versus sham group; (**E**) Representative images show double-labeling immunofluorescence of BrdU and GFAP positive cells in the hippocampal DG. There were no differences among the groups in the number of BrdU^+^/GFAP^+^ cells. Scale bar = 100 µm; (**F**) Bar graph represents the number of BrdU^+^/GFAP^+^ cells in the SGZ/GCL; (**G**) Bar graph show the proportion of BrdU^+^/GFAP^+^ cells in BrdU^+^ cells of the SGZ/GCL. Data are means ± SE, *n* = 5–8 from each group. * *p* < 0.05, versus sham group.

We then investigated the phenotype of cells that have survived in the late phase of STZ-induced diabetes. The phenotypes of newly proliferated cells in the DG include neuron (BrdU^+^/NeuN^+^) and glia (BrdU^+^/GFAP^+^). Numerous BrdU positive cells were also positive for NeuN. The proportion and number of BrdU positive cells expressing NeuN was significantly decreased in the STZ-injected group compared to the sham-operated group. However, no significant differences in the proportion and number of BrdU positive cells expressing NeuN were found between vehicle- and ZC-treated rats under sham-operation or STZ injection. In addition, a significant difference was not observed in the number of BrdU positive cells expressing GFAP between the groups. However, the proportion of BrdU positive cells expressing GFAP was significantly increased in the STZ-injected group compared to the sham-operated group (Figure 5). These results suggest that ZC treatment has no effect on neurogenesis during the late phase of STZ-induced diabetes.

## 3. Discussion

The effects of zinc supplementation on hippocampal neurogenesis in diabetes mellitus have not been evaluated previously in rat. Hippocampal neurogenesis was assessed by BrdU, Ki67, and DCX immunohistochemistry at 10 (early phase) or 45 (late phase) days after STZ injection. In the present study, we found that daily zinc supplemented with a histidine/proline complex given during the early phase of diabetes increased progenitor cell proliferation and neuroblast production in diabetic rats. However, long-term zinc supplement in the late phase of diabetes showed no neurogenic effects in diabetic rats.

Patients suffering from uncontrolled diabetes suffer from various forms of cognitive impairment [1,2,3,18]. Several behavioral studies in diabetic rats have shown mixed results, possibly due to the different diabetes models used. For instance, different degrees or forms of stress arising from different behavioral tests may account for some variability. Larger behavioral problems are associated with the STZ-induced diabetic model than in non-diabetic rats or other diabetic animal models [19]. STZ-diabetic rodents can perform easy and basic tasks [20], however, if more-complicated tasks are employed, STZ-diabetic rats show apparent cognitive impairment [20]. As in chronic diabetic patients, a diverse number of cognitive functions worsen in diabetic rats. A reduction in conduction velocity was described initially in the peripheral nervous system and later in the central nervous system [21,22]. Visual and auditory problems arise at only three to four months after the onset of diabetes and steadily worsen thereafter [22,23]. The precise mechanism underlying cognitive dysfunction in diabetic patients remains unclear.

Synaptic plasticity is thought to supply a cellular basis for cognitive function in the brain [24]. In particular, the hippocampus is an important center for spatial memory in rodents. Several groups have demonstrated that hippocampal synaptic strength is weakened in diabetic rats, which leads to functional cognitive impairment. Accordingly, a reduction in synaptic strength has been reported in hippocampal slice experiments from STZ-induced diabetic rats [19]. Mounting data suggest that newly born cells from the subgranular zone of the hippocampus mature and functionally integrate into the DG. These newly developed cells display normal physiological parameters such as receptive membrane properties and action potentials, behaving like mature DG cells [25]. Newly generated neurons from the DG play a leading part in synaptic plasticity [26]. Reduction in the number or functional disintegration of newly generated cells worsens learning and memory [9]. Neurogenesis after transient cerebral ischemia not only leads to the replacing of injured cells, but also influences functional recovery [27,28]. Our previous study also demonstrated that hypoglycemia, seizure, or traumatic brain injury-induced cognitive impairment also display reduced neurogenesis in the hippocampus. We further suggested that divalent zinc is associated with injury-induced neurogenesis. Therefore, these results further support the concept that strategies for increasing endogenous neurogenesis may hold potential for the development of restorable therapy [11].

Ionic zinc is the second most plentiful transition element in the brain, following iron. Chelatable zinc is highly localized in the synaptic vesicle of mossy fiber terminals of the hippocampus and in the olfactory blub [29], areas where neurogenesis and neural migration actively occur in the adult brain [30]. Zinc ion is considered to be a biologically essential element for both systemic physiology and brain function. This ion is a constituent of over 1000 enzymes and many transcription factors, including zinc finger proteins. Thus, ionic zinc is involved in a truly broad diversity of cellular functions such as DNA synthesis and cell division [31]. Zinc also affects humoral control of cell division by pituitary growth hormone including nerve growth factor (NGF) [32] or insulin-like growth factor (IGF) [31]. Cerebellar granular cell division and migration was also impaired after severe zinc deficiency [33,34,35]. Reduced dietary zinc also impaired performance in short-term memory tasks [36]. The above evidence demonstrates that zinc is an essential element required for development, cell division, migration, and proliferation, and further suggests that this metal ion may have a vital role in cognitive function and neurogenesis [37].

Streptozotocin (STZ)-induced diabetic rats cannot secrete new insulin because of damage to the beta cells of the pancreatic islet. STZ administration induces a rapid breakdown of the beta cells, causes hyperglycemia [38], and produces metal ion dysmetabolism [39]. However, if the dose of STZ is not sufficient for complete destruction of the islet cells, new islet cells can be regenerated [40]. The zinc supplement compound cyclo-(His-Pro) (ZC) showed a reduction of blood glucose concentration in streptozotocin-induced diabetic rats, likely via stimulation of glucose uptake by the action of zinc on the b-subunit of insulin receptor [41,42]. Although ZC may have separate effects at the level of the insulin receptor or glucose transporter, it is very likely that they positively work on blood glucose control in diabetes patients via activating zinc metabolism [16]. It is not clear to what degree ZC’s influence on blood glucose metabolism is directly linked to the action of zinc transporters, passive transport, or cation exchange [43]. Although previous studies showed a reduction of blood glucose levels by ZC administration, the present study showed no difference in blood glucose concentrations between the ZC treated group and the vehicle treated group. We cannot explain this difference. However, we can speculate that our STZ-induced model of diabetes may have been too severe, since over-administration of STZ not only destroys pancreas beta cells but may also inhibit insulin receptors [44] or glucose transporters [45] at high doses. This is one possibility to explain why ZC treatment in the present study had no ability to reduce blood glucose levels. Thus, the present study showed that ZC and vehicle-treated groups had similar blood glucose levels in the STZ-induced diabetic rat. Even though blood glucose levels were not affected by ZC administration, proliferation and differentiation were increased by ZC administration during the early phase of STZ-induced diabetes However, during the late phase neural differentiation was not increased by ZC administration. These results suggest that conditions that impose a state of chronic hyperglycemia may deleteriously affect neural differentiation. However, the exact mechanism should be confirmed by future studies.

Zinc influx and outflow is modulated by an active transport process that promotes homeostasis and prevents zinc toxicity. ZC enhances zinc transport from the intestinal lumen into the enterocytes [46]. ZC is a cyclic form of l-histidine and l-proline amino acid complex. Histidine and proline are highly distributed throughout the human body [45]. These amino acids are a permanent metabolite of both thyrotropin-releasing hormone [47] and histidine-proline-rich glycoproteins [48]. This glycoprotein is responsible for zinc transport from the intestine to body. The ZC motif, similar in sequence to specific zinc transporters, in the intestine facilitates zinc transport into human cells [49]. ZC is also found in high concentrations in the brain after administration [50]. Song et al. has reported that ZC is an efficient compound to increase zinc metabolism in the body [15]. Furthermore, our recent study demonstrated that zinc supplementation by ZC treatment increases hippocampal neurogenesis and levels of vesicular zinc [14]. Therefore, the present study suggests that ZC improves zinc absorption into the brain and has a beneficial effect on hippocampal neurogenesis.

## 4. Materials and Methods

### 4.1. Ethics Statement

This study was performed by the Guide for the Care and Use of Laboratory Animals of the National Institutes of Health (NIH). Use of animals in this study was approved by the Committee on Animal Use for Research and Education at Hallym University (Protocol # Hallym 2015-48).

### 4.2. Experimental Animals

Juvenile Sprague-Dawley male rats (90–100 g), aged four weeks, were purchased from DBL (DBL Co., Chungcheongbuk, Korea). The animals were kept in a temperature and humidity controlled room (22 ± 2 °C, 55% ± 5%, and a 12 h light: 12 h dark cycle), supplied with Purina diet (Purina, Gyeonggi, Korea) and water ad libitum. All animals were adapted for one week to avoid stress associated with transportation.

### 4.3. Rat Model of Type 1 Diabetes

For induction of type 1 diabetes, rats were intraperitoneally injected with streptozotocin (STZ, 50 mg/kg, IP) once per day for two consecutive days. STZ powder was dissolved in 0.1M sodium citrate buffer (pH = 4.5). In the present study we defined diabetes as overnight fasting tail blood glucose levels in excess of 200 mg/dL 24 h after the STZ injection. We found that most animals receiving STZ showed typical diabetes symptoms as seen in previous studies [51]. Tail blood glucose level was measured using a One Touch Basic Glucometer (Accu-Chek Active, Roche, Germany) [52].

### 4.4. Zinc Supplementation

For zinc supplementation, we used zinc plus cyclo-(His-Pro) (zinc plus CHP, ZC), formulated as a gel capsule containing 200 mg bovine prostate powder supplemented with 20 mg zinc [53]. ZC (27 mg/kg) was administered by gavage once daily for one or six weeks from the third day after the STZ injection, and then brains were harvested at 10 or 45 days, respectively. The vehicle-treated group was fed cyclo-(His-Pro) without zinc for the same periods. Animals were randomly divided into four groups to evaluate the effects of ZC treatment for early phase: (1) vehicle-treated sham group (Sham + Vehicle, *n =* 12); (2) ZC-treated sham group (Sham + ZC; *n* = 7); (3) vehicle-treated STZ group (STZ + Vehicle, *n* = 12); and (4) ZC-treated STZ group (STZ + ZC, *n* = 10). In the early phase, STZ-induced diabetic rats underwent hyperglycemia for seven days. Next, animals were randomly divided into four groups to evaluate the effects of ZC treatment for late phase: (1) vehicle-treated sham group (Sham + Vehicle, *n* = 7); (2) ZC-treated sham group (Sham + ZC; *n* = 8); (3) vehicle-treated STZ group (STZ + Vehicle, *n* = 7); and (4) ZC-treated STZ group (STZ + ZC, *n* = 6). In the late phase, STZ-induced diabetic rats underwent hyperglycemia for 42 days.

### 4.5. BrdU Labeling

To test the effects of zinc supplementation by ZC on neurogenesis in diabetic rats, 5-Bromo-2-Deoxyuridine (BrdU, 50 mg/kg; Sigma, St. Louis, MO, USA) was injected twice daily for four consecutive days starting the sixth day after the STZ injection. The rats were sacrificed at either 10 or 45 days after first STZ injection.

### 4.6. Brain Sections Preparation

To fix the brain, rats were anesthetized by overdose of intraperitoneal injection of urethane (1.5 g/kg, IP). For the blood wash out from the brain, 0.9% normal saline was perfused through heart for 10 min and then switched by 4% paraformaldehyde (PFA) in 0.1 M phosphate buffered saline (PBS, pH 7.4). Thirty minutes after PFA perfusion, the brains were taken out and post-fixed in the 4% PFA for one hour. After then, the brains were cryoprotected by 30% sucrose solution for overnight. When the brains were sank at the bottom, the entire brain was frozen by powered dry ice. After then, the brains were cut with Leica CM1850 cryostat (Leica Biosystems, Wetzlar, Germany) at 30 µm thickness.

### 4.7. Immunohistochemistry

Frozen sections were incubated in 0.6% H_2_O_2_ for 15 min at room temperature and washed three times with PBS. For immunohistochemical staining, mouse anti-BrdU (1:150, Roche, Basel, Switzerland), rabbit anti-Ki67 (1:1000, Novocastra, UK), or guinea pig anti-DCX (1:2000, Millipore, Billerica, MA, USA), diluted in PBS containing 0.3% normal chicken serum and 0.3%Triton X-100, were used as the primary antibodies and incubated overnight at 4 °C. The sections were washed three times for 10 min each with PBS, incubated in biotinylated anti-mouse, anti-rabbit or anti-guinea pig IgG (1:250, Vector Laboratories, Burlingame, CA, USA), and then avidin-biotinylated enzyme complex (ABC reagent, Vector Laboratories), and diluted 1:250 in the same solution as the primary antiserum. The immunoreactivity was revealed with 3,3′-diaminobenzidine (DAB, Sigma-Aldrich Co., St. Louis, MO, USA) in 0.01 M PBS buffer and mounted on the gelatin-coated slides.

### 4.8. Immunofluorescence Staining

For BrdU and Nestin double immunostaining in the early phase, sections were immersed with 2 N HCl at 37 °C for 90 min, neutralized two times for 10 min with 0.1 M sodium borate buffer, and incubated in a mixture of rat monoclonal anti-BrdU (1:150, Abcam, Cambridge, UK) and mouse monoclonal anti-Nestin (1:200, Abcam) for 2 h at room temperature. After rinse with PBS, the sections were incubated in a mixture of Alexa Fluor 488 donkey anti-mouse IgG (Nestin) and Alexa Fluor 594 donkey anti-rat IgG (BrdU) antibodies (1:250, Invitrogen, Grand Island, NY, USA) for 2 h at room temperature. To determine the phenotype of newly generated cells, double immunofluorescent labeling with BrdU and either NeuN or GFAP was performed in mice of the late phase (36 days after the last BrdU injection). Sections were incubated for 2 h in a mixture of mouse monoclonal anti-BrdU (1:150, Roche) and either rabbit polyclonal anti-NeuN (neuronal nuclei, 1:500, Millipore) or goat polyclonal anti-GFAP (glial fibrillary acidic protein, 1:200, Abcam) followed by 2 h of incubation in a mixture of Alexa Fluor 594 donkey anti-mouse IgG (BrdU) and either Alex Fluor 488 donkey anti-rabbit IgG (NeuN) or donkey anti-goat (GFAP) antibodies (1:250, Invitrogen) for 2 h at room temperature. Fluorescence signals were detected using a Zeiss LSM 710 confocal imaging system (Carl Zeiss, Oberkochen, Germany) with a sequential scanning mode for Alexa 488 and 594. Stacks of images (1024 × 1024 pixels) from consecutive slices of 0.9–1.2 µm in thickness were obtained by averaging eight scans per slice and were processed with ZEN 2010 (Carl Zeiss).

### 4.9. Quantification

To quantify BrdU, Ki67, and DCX-positive cells, sections were collected from 2.8 to 4.5 mm posterior to bregma and five coronal sections were analyzed from each animal. An experimenter masked to the treatment condition counted the number of BrdU, Ki67, and DCX-positive cells in the subgranular zone (SGZ) and granular cell layer (GCL) of dentate gyrus from both hemispheres. The mean numbers of BrdU, Ki67, and DCX-positive cells were used for statistical analyses. To analyze the phenotype of BrdU-positive cells, we determined whether BrdU-positive cells in the SGZ and GCL expressed Nestin, NeuN or GFAP with confocal microscopy. A double positive percentage was calculated as BrdU^+^/NeuN^+^ or BrdU^+^/GFAP^+^ cells for total BrdU-positive cells.

### 4.10. Data Analysis

Data are shown as mean + SEM. Statistical significance was assessed by analysis of variance (ANOVA) followed by the Student-Newman-Keuls *post-hoc* test. *p*-values less than 0.05 were considered statistically significant.

## 5. Conclusions

In the present study, we found that progenitor cell proliferation in the hippocampus of diabetic rats showed an increase in the early phase after STZ injection. Further, we found that ZC treatment significantly increased NPCs proliferation and neuroblast production in the early phase of STZ-induced diabetic rats. In addition, ZC treatment showed increased survival rate of newly generated cells, but no difference in the level of neurogenesis in the late phase of STZ-induced diabetic rats. Taken together, the present study suggests that zinc supplementation by ZC increases both NPCs proliferation and neuroblast production at the early phase of diabetes. Thus, this study suggests that zinc supplemented with a histidine/proline complex may have beneficial effects on neurogenesis in diabetic patients.

## Figures and Tables

**Table 1:** Effect of zinc plus cyclo-(His-Pro) (ZC) on change in body weight of sham and streptozotocin (STZ)-induced diabetic rats. Values are means ± SE, *n* = 6–12 from each group; * *p* < 0.05, versus sham group.

**Groups**	**Body Weight (g)**	
	**10 Days**	
	**Initial**	**Final**
Sham + Vehicle (*n* = 12)	177.17 ± 1.89	224.75 ± 3.39
Sham + ZC (*n* = 7)	167.57 ± 5.36	227.29 ± 6.89
STZ + Vehicle (*n* = 12)	172.75 ± 3.08	165.92 ± 5.09 *
STZ + ZC (*n* = 10)	166.40 ± 1.56	162.00 ± 2.71 *
**Groups**	**Body Weight (g)**	
	**45 Days**	
	**Initial**	**Final**
Sham + Vehicle (*n* = 7)	133.14 ± 4.34	405.00 ± 7.51
Sham + ZC (*n* = 8)	127.00 ± 2.42	387.75 ± 9.66
STZ + Vehicle (*n* = 7)	133.20 ± 5.34	213.60 ± 24.41 *
STZ + ZC (*n* = 6)	132.60 ± 2.52	184.40 ± 13.08 *

**Table 2:** Effect of ZC on change in blood glucose level of sham and STZ-induced diabetic rats. Values are means ± SE, *n* = 6–12 from each group; * *p* < 0.05, versus sham group.

**Groups**	**Blood Glucose Level (mg/dL)**	
	**10 Days**	
	**Initial**	**Final**
Sham + Vehicle (*n* = 12)	116.92 ± 4.39	115.83 ± 4.04
Sham + ZC (*n* = 7)	117.29 ± 5.85	114.00 ± 4.48
STZ + Vehicle (*n* = 12)	112.75 ± 3.67	518.08 ± 17.24 *
STZ + ZC (*n* = 10)	115.90 ± 3.64	488.50 ± 34.20 *
**Groups**	**Blood Glucose Level (mg/dL)**	
	**45 Days**	
	**Initial**	**Final**
Sham + Vehicle (*n* = 7)	110.29 ± 3.63	90.57 ± 3.73
Sham + ZC (*n* = 8)	109.63 ± 4.74	87.00 ± 3.35
STZ + Vehicle (*n* = 7)	105.00 ± 3.78	551.60 ± 20.99 *
STZ + ZC (*n* = 6)	105.40 ± 5.10	562.80 ± 16.92 *

## Abbreviations

ZC	Zinc plus cyclo-(His-Pro)
DG	Dentate gyrus
DCX	Doublecortin
STZ	Streptozotocin
GCL	Granular cell layer
SGZ	Subgranular zone
SVZ	Subventricular zone
NPCs	Neural progenitor cells
BrdU	5-Bromo-2-Deoxyuridine
PFA	Paraformaldehyde
PB	Phosphate buffer
ABC	Avidin-biotinylated enzyme complex
DAB	3,3′-Diaminobenzidine
NeuN	Neuronal nuclei
GFAP	Glial fibrillary acidic protein
IP	Intraperitoneal
PO	Per os
PBS	Phosphate-buffered saline
